# A novel VARS2 gene variant in a patient with epileptic encephalopathy

**DOI:** 10.1080/03009734.2019.1670297

**Published:** 2019-10-18

**Authors:** Lucija Ruzman, Ivana Kolic, Jelena Radic Nisevic, Antonija Ruzic Barsic, Ingrid Skarpa Prpic, Igor Prpic

**Affiliations:** a Child Neurology and Child Psychiatry Department, Pediatric Clinic, Clinical Hospital Center Rijeka, Rijeka, Croatia;; b University of Rijeka, School of Medicine Rijeka, Rijeka, Croatia;; c Radiology Department, Thalassoterapia Opatija, Opatija, Croatia;; d Neurology Clinic, Clinical Hospital Center Rijeka, Rijeka, Croatia

**Keywords:** Encephalocardiomyopathy, epileptic encephalopathy, mitochondrial disease, VARS2

## Abstract

**Background:** Mitochondrial disorders are heterogeneous clinical syndromes caused by defective activity in the mitochondrial respiratory chain, resulting in a faulty oxidative phosphorylation system. These inherited disorders are individually rare, and furthermore they are phenotypic variables. The genetically characterized mitochondrial disorders are rarely associated with epileptic encephalopathies.

**Case presentation:** We present the clinical phenotype, biochemical analysis, and electrographic and neuro-radiological features of a 5-month-old girl with epileptic encephalopathy, microcephaly, severe psychomotor delay, hypertrophic cardiomyopathy, and abnormal MRI scan. Using whole-genome sequencing technique, compound heterozygous mutations of the VARS2 gene were revealed, with one previously unreported frameshift mutation.

**Conclusion:** Our report extends the phenotypic spectrum of VARS2-related disorders with an initial presentation of epileptic encephalopathy and early death due to malignant arrhythmia.

## Introduction

Mitochondrial disorders are heterogeneous clinical syndromes caused by defective activity in the mitochondrial respiratory chain (MRC), resulting in a faulty oxidative phosphorylation system (OXPHOS) (1), associated with a broad range of nuclear and mitochondrial gene mutations. These inherited disorders are rare and phenotypically variable, and thus generally unfamiliar to clinicians. Epileptic encephalopathies (EE) constitute a heterogeneous group of disorders characterized by intractable seizures with specific abnormalities on electroencephalography (EEG) and severe developmental delay or regression (2); however, a clearly defined aetiology and a diagnostic algorithm for these clinical entities have not been fully established.

In our case study we present a clinical phenotype, a biochemical analysis, as well as electrographic and neuroradiological features of a patient with EE. A genetic analysis revealed an MRC defect.

## Methods

### Acquisition of a clinical case

We made a comprehensive evaluation of our patient, including molecular genetics investigations. The patient’s parents signed an informed consent in agreement with the Declaration of Helsinki.

### Molecular genetics

Genomic DNA from the proband, father, and mother was isolated from peripheral blood samples, and whole-exome sequencing (WES) was completed. WES, bioinformatic analysis, variant confirmation, and segregation analysis was performed in the CeGaT GmbH laboratory (Tübingen, Germany). Coding and flanking intronic regions were enriched using the Agilent in solution technology and were sequenced using the Illumina HiSeq/NovaSeq system (Illumina Inc., San Diego, California, USA). Variants (SNVs/small indels) with a minor allele frequency (MAF) <1.5% were evaluated. Known disease-causing variants (according to HGMD) were evaluated up to 5% MAF. The MAF were taken from the 1000 Genomes, dsSNP, GnomAD, and in-house database. For the index case, 95.61% of the targeted regions were covered by a minimum of 30 high-quality sequencing reads per base. All filtered variants were further analysed using Mutation Taster, fathmm, Mutation Assessor, SIFT, fathmm-MKL coding, LRT, and PROVEAN for pathogenicity prediction. Two algorithms were applied (3) for assessments of the consequences of variants on splicing. WES identified compound heterozygous mutations in the VARS2 gene (NM_001167734.1) in our patient. Both parents are heterozygous carriers of variants in VARS2.

## Results

A female child was born at term after an uneventful pregnancy and delivery. She was the first child from the first pregnancy of healthy non-consanguineous parents. The family history was unremarkable. The birth weight, length, and head circumference were in accordance with the gestational age (4030 g, 51 cm, and 34 cm, respectively). Computed tomography (CT) was performed at one month of age because of failure of head growth (50th percentile at birth and 10th after neonatal period). Craniosynostosis was excluded, with no other pathological findings. Neurohabilitation was started due to delayed acquisition of milestones.

**Figure 1.**
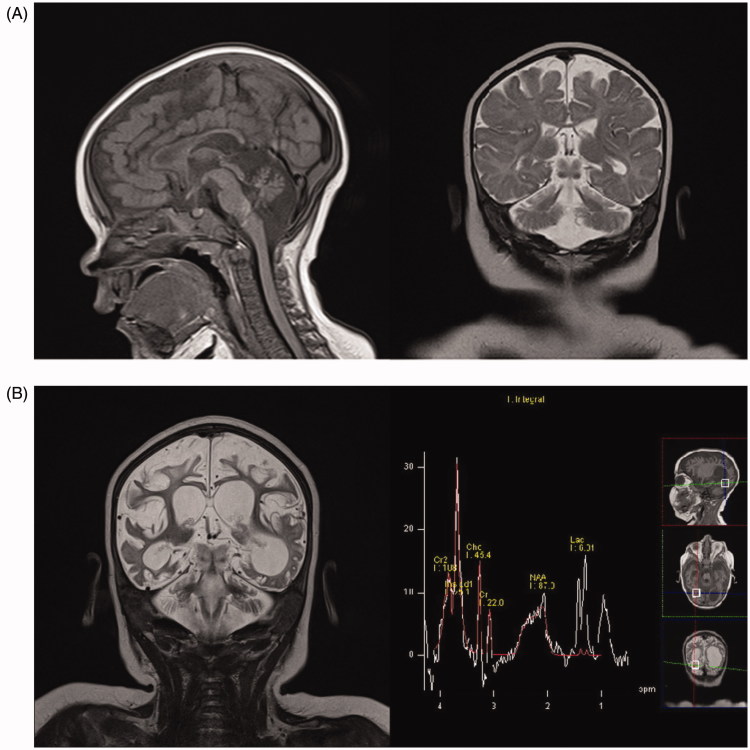
(A) First MRI scan, age 5 months. Sagittal T1-weighted and coronal T2-weighted imaging showing diffuse white matter reduction, marked cerebral and cerebellar atrophy, corpus callosum thinning, and brainstem hypoplasia. (B) MRI scan, age 9 months. Coronal T2-weighted imaging showing marked progression of cerebral and cerebellar atrophy and ventriculomegaly (dominantly left occipital ventricle) due to diffuse white matter necrosis, while MRI spectroscopy showed markedly increased myoinositol peak, increased lactate peak, and reduced N-acetyl aspartate peak.

At 5 months of age she developed infantile spasms consistent with hypsarrhythmia on EEG studies. Clinical examinations showed microcephaly with ‘pear-shaped face’, hypertelorism, and a wide nasal bridge. Global motor development was delayed by 2–3 months. Language and social skills were in accordance with the patient’s age. Laboratory findings revealed an increased concentration of alanine in plasma (537 μmol/L, normal range 100–439 μmol/L) and cerebrospinal fluid (36.1 μmol/L, normal range 19.9–31.3 μmol/L), as well as mildly elevated concentrations of lactate in plasma (2.0–5.4 mmol/L, normal range 0.33–2.0 mmol/L) and cerebrospinal fluid (3.0 mmol/L, normal range 1.1–2.8 mmol/) indicative of mitochondrial disorder, while others were normal. Magnetic resonance imaging (MRI) performed at that time revealed diffuse cerebral atrophy with more prominent hypoplasia of the cerebellum (especially vermis), brainstem, and corpus callosum (Figure 1(A)).

At the age of 6 months multiple seizure types (mastication, eyelid and lip twitching, myoclonic seizures of the trunk and extremities, tonic and clonic seizures) were noticed. Treatment attempts with several antiepileptic drugs including folic acid, cholecalciferol, pyridoxine and modified Atkins diet were not successful. EEG studies showed continuous burst–suppression pattern. All in all, the clinical status of the child developed into EE. Microcephaly, severe global hypotonia, failure to thrive, and feeding and breathing difficulties were more and more pronounced. Pendular nystagmus and oedema of hands and feet were periodically noticed. Echocardiography showed hypertrophic obstructive cardiomyopathy with fast progression, and atenolol treatment was initiated.

Genetic analysis was performed at the age of 9 months, and it revealed compound heterozygous mutations of the VARS2 gene: previously reported pathogenic variant (c.1100C>T; p.Thr367Ile) transmitted from the father and one unreported frameshift mutation (c.603_606dupGATG; p.Arg203Aspfs*37) transmitted from the mother. An additional MRI with spectroscopy showed a further significant loss of the brain parenchyma and a high lactate peak (Figure 1(B)).

A multidisciplinary treatment approach was applied to treat the neurologic, cardiac, respiratory, and gastrointestinal complications. In accordance with the established diagnosis, treatment with L-carnitine and coenzyme Q10 was initiated. Despite multiple supportive and symptomatic treatment regimens, the child died of malignant arrhythmia at the age of 10 months.

## Discussion

Combined oxidative phosphorylation deficiency 20 (COXPD20) (OMIM #615917) is an autosomal recessive mitochondrial encephalocardiomyopathy caused by variants in the VARS2 gene (OMIM #612802) located on chromosome 6p21 (https://www.omim.org/entry/615917). The VARS2 gene contains 30 exons and encodes mitochondrial valyl tRNA-synthetase, which participates in mitochondrial protein synthesis (4). Despite the estimated prevalence of MRC defects being 1 in 5000 live births, it is often difficult to find the molecular basis. The underlying genetic basis is not possible to identify in 40% to 70% of the patients, even though there is biochemical evidence of MRC defects (5). Presently, there are 10 reported pathogenic variants in the VARS2 gene (https://www.ncbi.nlm.nih.gov/clinvar/?term=VARS2[all]).

When considering our patient in comparison with previously reported patients with VARS2 mutations (5–10), it appears that the main phenotypic features are seizures, various degrees of developmental delay, facial dysmorphism, brain atrophy, and cardiomyopathy. The main clinical features, biochemical studies, and molecular findings are shown in Table 1.

**Table 1.** Clinical, biochemical and molecular findings in previously reported patients with VARS2 mutation.

	Our case	Diodato et al. (6); Pereira et al. (7); Bruni et al. (10)	Pronicka et al. (9)	Bruni et al. (10)	Taylor et al. (5)	Baertling et al. (8)	Bruni et al. (10)	Bruni et al. (10)	Bruni et al. (10)	Bruni et al. (10)
Variant in VARS2 gene	c.1100C>T; p.Thr367Ile c.603_606dupGATG; p.Arg203Asp*f*s*37	c.1100C>T; p.Thr367Ile	c.1100C>T; p.Thr367Ile c.1490G>A; Arg497His	c.1258G>A; p.Ala420Thr	c.1135G>A; p.Ala379Thr c.1877C>A; p.Ala626Asp	c.601C>T; p.Arg201Trp c.1100C>T; p.Thr367Ile	c.2557-2A>G; aberrant splicing c.1100C>T; p.Thr367Ile	c.1546G>T; p.Glu516* c.2239G>A; p.Ala747Thr	c.1100C>T; p.Thr367Ile c.1150G>A; p.Asp384Asn	c.1100C>T; p.Thr367Ile c.1490G>A; pArg497Hys
Sex	Female	Female (*n* = 4), Male (*n* = 2)	Male	Female (*n* = 1), Male (*n* = 2)	Male	Male	Female	Male	Female	Male
Age of onset	5 months	Neonatal period	Neonatal period	Neonatal period	<1 year	Neonatal period	Neonatal period	Neonatal period	3 months	Neonatal period
First symptom	Seizures (infantile spasms)	Neurological symptoms (seizures, hypotonia, and/or DD)	Stridor and respiratory failure	Hypotonia, seizures, and feeding difficulties	Hypotonia	Lactacidosis and respiratory failure	Stridor and apnoea	Stridor and poor feeding	Seizures	Stridor, hypotonia, and respiratory failure
Neurological symptoms	Seizures, hypotonia, DD, pendular nystagmus	Seizures, DD, spastic tetraparesis, and/or pendular nystagmus	Hypotonia, limb spasticity, seizures	DD, severe hypotonia, seizures	Hypotonia, ataxia, seizures	Spastic movement disorder, seizures	Hypotonia, hyporeflexia, hyperekplexia, seizures	Hypotonia, DD	Hypotonia, DD	Hypotonia, absence of swallowing reflex
Facial dysmorphism	Present	Present or absent	N/P	N/P	N/P	N/P	Present	Present	N/P	N/P
Cardiac signs	HCM	HCM or absent cardiac signs	HCM	HCM	HCM	HCM	HCM, pericardial effusion	HCM	HCM	HCM
Additional clinical signs	MIC, oedema of hands and feet, failure to thrive, feeding and breathing difficulties	MIC, feedings difficulties, failure to thrive	Cryptorchidism, chronic pancreatitis	Hepatosplenomegaly	Progressive external ophthalmoplegia and ptosis	MIC	Congenital hip dislocation, MIC	MIC, laryngomalatia	Feeding difficulties, MIC	N/P
Laboratory tests	↑ Lac and Ala in plasma and CSF	↑ Ala in plasma	↑ Lac in plasma	N/P	N/P	↑ Lac in plasma	↑ Lac and Ala in CSF and plasma	↑ Lac in plasma	↑ Lac and Ala in plasma	↑ Lac in plasma
EEG	Epileptiform abnormalities	Epileptiform abnormalities	N/P	N/P	N/P	Epileptiform abnormalities	N/P	N/P	Epileptiform abnormalities	N/P
MRI features	Cerebral atrophy with hypoplasia of cerebellum, brainstem, and corpus callosum	Cerebral or cerebellar atrophy and/or corpus callosum hypoplasia	Cerebral atrophy and vermis hypoplasia	N/P	Symmetrical basal ganglia calcifications	Corpus callosum and cerebellar hypoplasia	Cerebral and cerebellar atrophy	N/P	Corpus callosum and cerebellar hypoplasia	N/P
Age of death	10 months	*n* = 4 died at age from 28 months to 8 years; *n* = 2 still alive	9 years	*n* = 2 died before 3 months of age; *n* = 1 still alive	Alive at age of 18 years	N/P	3.5 months	19 months	5 months	3 months

Ala: alanine; CSF: cerebrospinal fluid; DD: developmental delay; HCM: hypertrophic cardiomyopathy; Lac: lactate; MIC: microcephaly; N/P: information not provided by the author.

Bruni et al. (10) elaborated clinical, biochemical, and molecular features in 13 patients from 9 families diagnosed by WES. Two previously reported cases by Taylor et al. (5) and by Pronicka et al. (9) were also included. Bruni et al. (10) presented a new homozygous (c.1258G>A; p.Ala420Thr) variant and four new compound heterozygous variants, along with already known pathogenic variants (Table 1). The study found the mutant VARS2 allele c.1100C>T; p.Thr367Ile the most common—in six patients in homozygous and in five patients in heterozygous states. Laboratory findings implied a mitochondrial disease, while the EEG and brain MRI of these patients did not show any disease-characteristic pattern. The common problem in these patients (*n* = 11/17) is hypertrophic cardiomyopathy, with only a few patients not being affected. The c.1100C>T; p.Thr367Ile variant is considered to have less of an effect on the heart (10). Alsemari et al. (11) published a study describing four patients with Angelman-like syndrome and mutation in the VARS2 gene, providing new insight into genotype–phenotype correlation.

To date, our case is one of the 17 described cases of severe infantile mitochondrial disorder associated with VARS2 pathogenic variants. The phenotypic spectrum of VARS2-related gene variants is complex, and no clear genotype–phenotype correlations have been established. Therefore, a detailed description of phenotype related to specific mutations is of great importance. Our report extends the phenotypic spectrum of VARS2-related disorders in the context of EE, severe neurodevelopmental delay, abnormal brain MRI scan, and one novel frameshift mutation (c.603_606dupGATG; p.Arg203Aspfs*37) in VARS2 (NM_001167734.1). With the exception of supportive care, currently no disease-modifying treatment exists for the disease.

The genetically characterized mitochondrial disorders are rarely associated with EE, with very few clinical reports available (12). A timely evaluation of patients with EE resulting in a specific diagnosis is of great value. We want to emphasize the importance of genetic analysis in the early diagnostic efforts of these patients. WES is a very powerful, non-invasive, accessible, and not overly expensive diagnostic tool. The finding of a specific gene mutation is of exceptional importance to clinicians for further management, and for the family to cope with the diagnosis and prognosis. Genetic counselling about familial risk, as well as for potential prenatal diagnostics, is of great value to the family.

Although the field of mitochondrial encephalomyopathies is highly dynamic, the genetic diagnosis of these disorders, which may offer the possibility to design targeted therapy, remains challenging. In the near future, the WES approach will provide valuable information regarding the genes implicated in MRC defects, which are important in the differential diagnosis of EE.
